# Effects of *ex vivo* Extracorporeal Membrane Oxygenation Circuits on Sequestration of Antimicrobial Agents

**DOI:** 10.3389/fmed.2021.748769

**Published:** 2021-12-01

**Authors:** Yuan Zhang, Hongbin Hu, Qing Zhang, Qing Ou, Huayou Zhou, Tong Sha, Zhenhua Zeng, Jie Wu, Jingrui Lu, Zhongqing Chen

**Affiliations:** ^1^Department of Critical Care Medicine, Nanfang Hospital, Southern Medical University, Guangzhou, China; ^2^Department of Pharmacy, Nanfang Hospital, Southern Medical University, Guangzhou, China; ^3^Department of Blood Transfusion, Nanfang Hospital, Southern Medical University, Guangzhou, China; ^4^Department of Mass Spectrometry, The Beijing Genomics Institute-Shenzhen, Shenzhen, China

**Keywords:** antimicrobial agents, extracorporeal membrane oxygenation, sequestration, *ex vivo*, intensive care unit

## Abstract

**Objectives:** Our *ex vivo* study was designed to determine the sequestration of teicoplanin, tigecycline, micafungin, meropenem, polymyxin B, caspofungin, cefoperazone sulbactam, and voriconazole in extracorporeal membrane oxygenation (ECMO) circuits.

**Methods:** Simulated closed-loop ECMO circuits were prepared using 2 types of blood-primed ECMO. After the circulation was stabilized, the study drugs were injected into the circuit. Blood samples were collected at 2, 5, 15, 30 min, 1, 3, 6, 12, and 24 h after injection. Drug concentrations were measured by high-performance liquid chromatography-tandem mass spectrometry. Control groups were stored at 4°C after 3, 6, 12, and 24 h immersing in a water bath at 37°C to observe spontaneous drug degradation.

**Results:** Twenty-six samples were analyzed. The average drug recoveries from the ECMO circuits and control groups at 24 h relative to baseline were 67 and 89% for teicoplanin, 100 and 145% for tigecycline, 67 and 99% for micafungin, 45 and 75% for meropenem, 62 and 60% for polymyxin B, 83 and 85% for caspofungin, 79 and 98% for cefoperazone, 75 and 87% for sulbactam, and 60 and 101% for voriconazole, respectively. Simple linear regression showed no significant correlation between lipophilicity (*r*^2^ = 0.008, *P* = 0.225) or the protein binding rate (*r*^2^ = 0.168, *P* = 0.479) of drugs and the extent of drug loss in the ECMO circuits.

**Conclusions:** In the two ECMO circuits, meropenem and voriconazole were significantly lost, cefoperazone was slightly lost, while tigecycline and caspofungin were not lost. Drugs with high lipophilicity were lost more in the Maquet circuit than in the Sorin circuit. This study needs more *in vivo* studies with larger samples for further confirmation, and it suggests that therapeutic drug concentration monitoring should be strongly considered during ECMO.

## Introduction

Extracorporeal membrane oxygenation (ECMO) is a prolonged form of cardiopulmonary bypass used to support patients with life-threatening respiratory or cardiac failure (1). The ECMO circuit consists of a membrane oxygenator, a centrifugal pump, a heat exchanger and, PVC tubing.

Patients receiving ECMO require multiple medications, including sedatives, analgesics, antimicrobial agents, anticoagulants, and vasoactive agents. The pharmacokinetics of drugs administered during ECMO is complicated. As an extension of the human cardiovascular system, the presence of ECMO circuits can further increase the total circulation volume, cause increases in the apparent volume of distribution (Vd), and lead to drug sequestration, thus affecting the pharmacokinetics of various drugs (2–4). *Ex vivo* experiments (5–7) confirmed significant drug sequestration in the ECMO circuit, and the extent of loss depends upon the physicochemical properties of the drug, the types of components of the circulation circuit, and circuit duration of use (8–10). Drugs with high octanol/water partition (log P), such as propofol (log P = 4.0) (8), have high solubility in organic materials and thus exhibit a considerable loss in the ECMO circuit.

Patients requiring ECMO treatment often have severe infectious diseases, so antimicrobial treatment is particularly critical. Inadequate antimicrobial treatment is closely associated with the presence of antibiotic resistance in clinically important pathogens (11) and may result in therapeutic failure. Some studies suggest a significant drug loss of meropenem (10), voriconazole (5), and caspofungin (6) within the ECMO circuits. However, the drug loss of teicoplanin, tigecycline, polymyxin B, and cefoperazone-sulbactam in ECMO circuits has not been reported. To address this issue, we set out to determine the drug absorption of these antimicrobial agents in different types of ECMO circuits. Our research attempts to provide experimental evidence for the use of teicoplanin, tigecycline, micafungin, meropenem, polymyxin B, caspofungin, cefoperazone sulbactam, and voriconazole in future ECMO treatment.

## Materials and Methods

### Ethics

This study was approved by the Medical Ethics Committee of NanFang Hospital of Southern Medical University (NFEC-2020-021). We obtained informed consent from each volunteer.

### Study Design and Participants

Four healthy volunteers were recruited. After obtaining informed consent, 402 ml of blood was collected through the cubitus vein using disposable blood bags (Fresenius Kabi), of which 2 ml was used for a routine blood examination. Sorin (LivaNova, London, United Kingdom) and Maquet (Getinge AB, Hirrlingen, Germany) ECMO circuits were used to establish self-circulation and were primed with fresh whole human blood. After the circulation stabilized, the drugs were added to the circuit. Blood samples were collected at different time points, and the drug concentration was measured to observe the recovery rate of different drugs in the ECMO cycle.

### Extracorporeal Membrane Oxygenation Circuits

**Figure 1:**
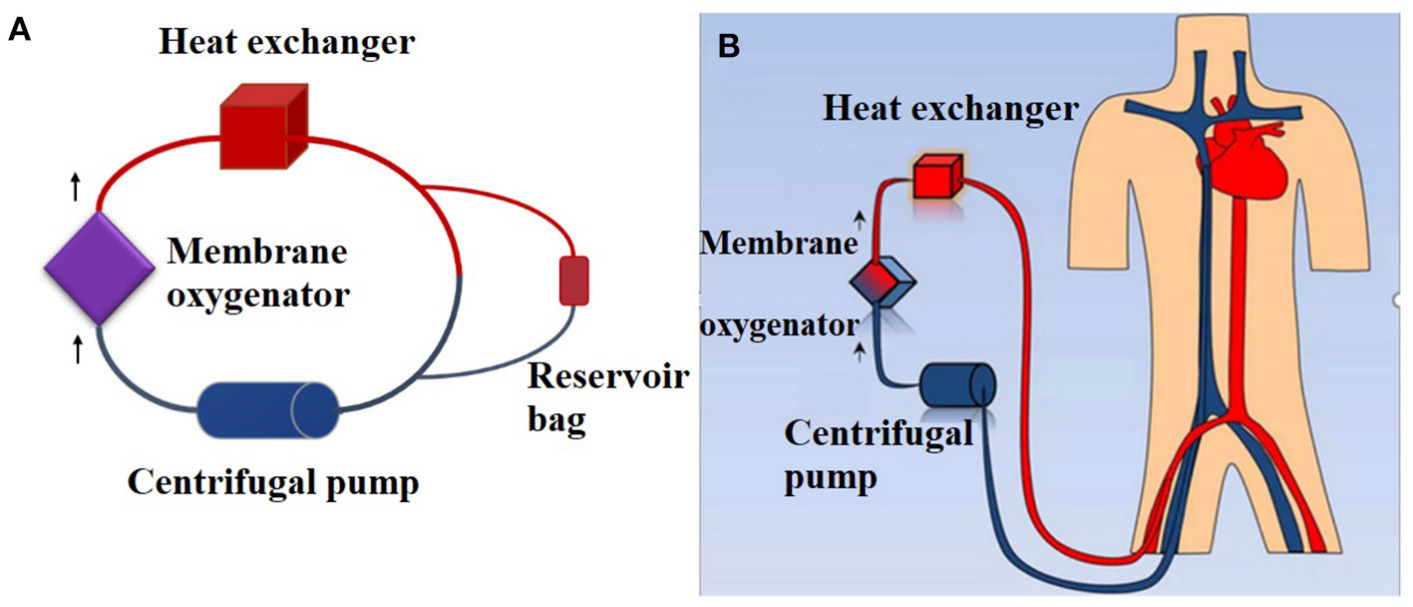
Simulated closed loop ECMO circuits primed with fresh whole human blood. **(A)** *Ex vivo* ECMO circuit. A reservoir bag containing 30 mL of blood was used to construct a bypass to maintain the pressure of the circuit. A total of 800 mL of fresh whole human blood (<1 h) was used to prime the circuit. **(B)** *In vivo* ECMO circuit.

Each circuit consisted of a membrane oxygenator, centrifugal pump, cannula, heat exchanger, and PVC tubing. The materials of each ECMO component are shown in Supplementary Material 1. A reservoir bag containing 30 mL of blood was used to construct a bypass to maintain the pressure of the circuit (Figure 1). Eight hundred milliliters of fresh whole human blood (<1 h old) was used to prime the circuit. Heparin (5,000 U) was added to the circuits to prevent clotting.

The final volumes in the Maquet circuit and Sorin circuit were 818 ± 1 mL and 525 ± 1 mL, respectively. The circuit flow rate was controlled at 4–5 L/min. Circuit temperature was maintained at 37°C. Carbon dioxide gas and sodium bicarbonate solution were added to the circuit to maintain the pH of the circulating blood in the range of 7.25–7.55.

### Drug Administration

When the temperature, flow rate, and pH of the ECMO circuits were stable, teicoplanin (120 mg), tigecycline (20 mg), micafungin (50 mg), meropenem (200 mg), polymyxin B (100,000 U), caspofungin (10 mg), cefoperazone-sulbactam (750 mg), and voriconazole (60 mg) were injected at 2-s intervals into a pre oxygenator injection site. These bolus doses were selected to produce concentrations similar to clinical concentrations. The order of administration was determined according to the half-life from long to short. According to the drug instructions, none of the drugs interact with each other (12). Two milliliters of physiological saline solution (0.9%) was used to flush the tube after injection of all drugs to avoid drug loss at the entrance of administration.

### Blood Sample Collection

Whole blood was collected in polypropylene tubes containing ethylenediaminetetraacetic acid (EDTA) from a post-oxygenator site and chilled to 4°C until further processing. Blood samples were collected from the ECMO operation group at 5, 15, 30 min, 1, 3, 6, 12, and 24 h after drug administration (13, 14). The control group did not pass through the circuits but was kept at the same warming temperature to observe the spontaneous degradation of the drug. Ten milliliters of blood were collected 2 min after the injection of all the drugs, of which 8 ml was divided into 4 tubes and stored at 4°C after 3, 6, 12, and 24 h of immersion in a water bath at 37°C separately as a control group. The blood drug concentration of the remaining 2 ml sample was regarded as the baseline value of all sampling points.

### Measurement of Drugs in Plasma Samples

All blood samples were stored at 4°C and centrifuged (10 min at 3,000 × g) within 8 h after sampling, and the plasma was separated and stored in clean polypropylene cryogenic vials at −80°C until analysis. The blood concentration of various drugs was measured through high-performance liquid chromatography-tandem mass spectrometry (HPLC-MS/MS). The recoveries of each drug after different times of circulation were calculated based on the blood concentration at 2 min.

Intra- and inter-assay means were within 15% of the target range value. For tigecycline, the linear calibration range was 0.07–8 ug/ml. For cefoperazone, the linear calibration range was 2.67–320 ug/ml. For sulbactam, the linear calibration range was 0.93–112 ug/ml. For teicoplanin, the linear calibration range was 1.6–192 ug/ml. For caspofungin, the linear calibration range was 0.53–64 ug/ml. For meropenem, the linear calibration range was 1–120 ug/ml. For voriconazole, the linear calibration range was 0.27–32 ug/ml. For micafungin, the linear calibration range was 0.54–64 ug/ml. For polymyxin B, the linear calibration range was 0.93–112 ug/ml.

Drug concentrations were measured using a Shimadzu (Kyoto, Japan) LC-20AD UHPLC system interfaced with a Shimadzu LCMS-8040 triple quadrupole mass spectrometer (MS/MS). Data acquisition and quantitative analysis were carried out using Shimadzu LabSolutions software.

### Statistical Analysis

Statistical analysis was performed using SPSS software for Windows, version 26 (IBM Corp, Armonk, NY, USA). Paired *t*-tests were used to compare the differences in drug recoveries at 24 h between the ECMO operation group and the control group. A *P*-value < 0.05 was considered to indicate statistical significance. The concentration-vs.-time curves (mean ± standard error of the mean) were plotted using GraphPad Prism version 8.0 (GraphPad Software, Inc., La Jolla, CA, USA). Log P and protein binding rate for the individual drugs were obtained from DrugBank^®^, a web-accessible public database (12). We used simple linear regression to explore the relationship between the log P or protein binding rate of drugs and the extent of their loss in the circuit at the end of 24 h.

## Results

The *ex vivo* circuits were maintained under physiological conditions for 24 h with no complications during ECMO operation. The pH value in the individual circuits over the 24 h was between 7.226 and 7.504 (Supplementary Material 2). The circuit flow rate was 4.25–4.74 L/min. In this study, two experiments were carried out and 26 blood samples were analyzed. The first experiment was conducted with the Maquet ECMO circuit. The drugs studied were teicoplanin, tigecycline, meropenem, caspofungin, cefoperazone-sulbactam, and voriconazole. The blood samples collected at each time point were measured twice due to the uncertainty of the initial experiment. The second experiment was carried out with Sorin ECMO circuit, micafungin and polymyxin B were added in addition to the above drugs, and the blood samples were measured only once.

### Drug Loss of Experimental Drugs in ECMO Circuits and Control Groups

**Figure 2:**
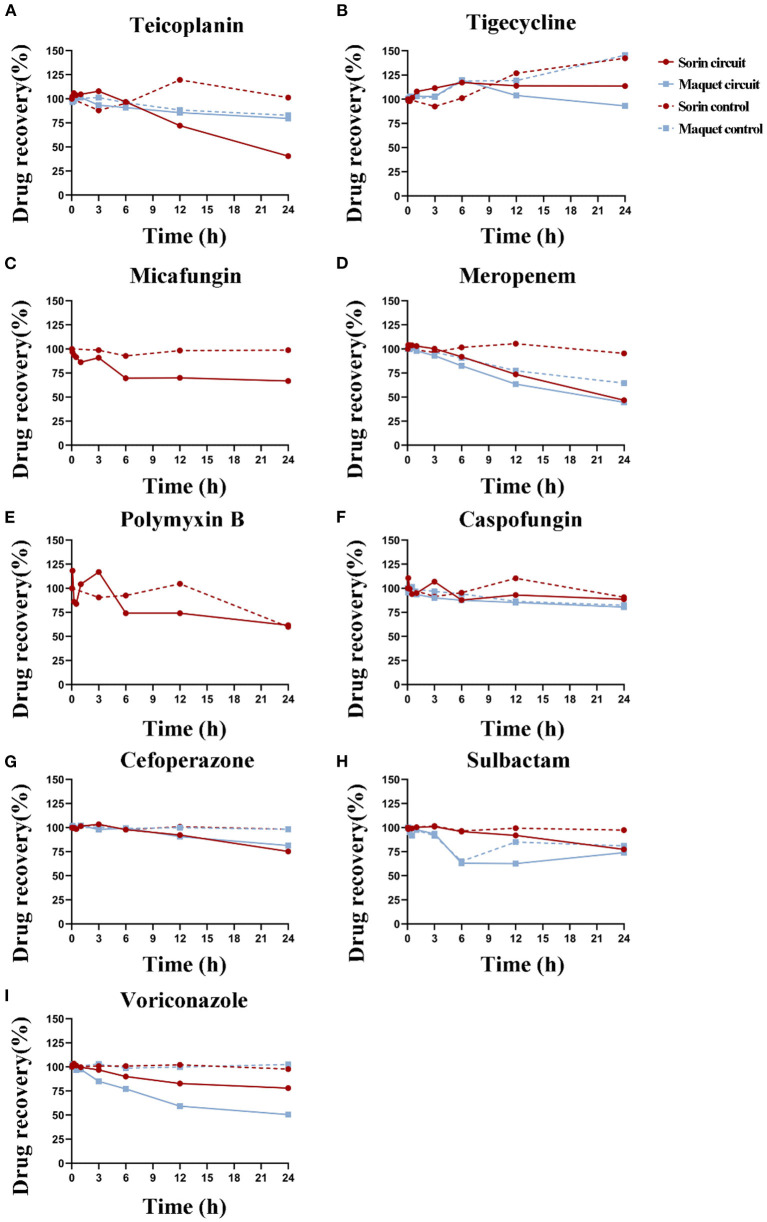
Durg recovery of experimental drugs at different time points in Sorin and Maquet ECMO circuits and corresponding control groups. Average drug recovery vs. time for **(A)** teicoplanin, **(B)** tigecycline, **(C)** micafungin, **(D)** meropenem, **(E)** polymyxin B, **(F)** caspofungin, **(G)** cefoperazone, **(H)** sulbactam, and **(I)** voriconazole in the Maquet (blue) and Sorin (red) circuits and corresponding control groups.

A total of 26 samples were analyzed. After 24 h of operation of the two types of ECMO circuits, significant drug loss occurred in meropenem and voriconazole, and a small loss in cefoperazone, while no significant loss was observed in tigecycline and caspofungin in both ECMO circuits (Figure 2). In the Sorin circuit, significant drug loss occurred in teicoplanin, micafungin, and polymyxin B, while a small amount of drug loss occurred in sulbactam. There was no significant difference in the recovery rates of teicoplanin and sulbactam between the Maquet circuit and the control group. In the Maquet circuit, drug loss for voriconazole (*P* = 0.018) and at 24 h was significantly higher than the drug loss in control groups. But there were no other significant differences in drug loss for meropenem (*P* = 0.301), tigecycline (*P* = 0.100), caspofungin (*P* = 0.559), sulbactam (*P* = 0.105), cefoperazone (*P* = 0.079) and teicoplanin (*P* = 0.094) at 24 h between circuit and control group.

The average drug recoveries from the ECMO circuits and control groups at 24 h relative to baseline were 67 and 89% for teicoplanin, 100 and 145% for tigecycline, 67 and 99% for micafungin, 45 and 75% for meropenem, 62 and 60% for polymyxin B, 83 and 85% for caspofungin, 79 and 98% for cefoperazone, 75 and 87% for sulbactam, and 60 and 101% for voriconazole, respectively (Table 1). Detailed data on drug recovery for each drug at different time points in each circuit are shown in Supplementary Materials 3, 4.

**Table 1:** The log P*-*value and protein-binding rate of drugs and the extent of their loss in the blood-primed circuit at 24 h.

**Drug**	**Drug recovery (%) from circuits at 24 h**	**Drug recovery (%) from control group at 24 h**	**Lipophilicity ( log P)^†^**	**Protein binding rate (%)**
Teicoplanin	66.64 (24.37)	89.06 (14.40)	−1.1	90–95
Tigecycline	100.03 (13.68)	144.54 (2.31)	0.8	71–89
Micafungin	66.85 (-)^*^	98.82 (-)^*^	−1.5	99
Meropenem	45.37 (3.58)	74.84 (19.10)	−0.6	2
Polymyxin B	61.66 (-)^*^	60.10 (-)^*^	−4.861	79–92
Caspofungin	83.26 (6.17)	85.24 (7.93)	−2.798	97
Cefoperazone	79.41 (19.16)	98.30 (1.17)	−0.74	82–93
Sulbactam	75.15 (6.50)	86.55 (11.31)	−0.92	38
Voriconazole	59.70 (16.00)	101.01 (2.85)	2.561	58

*Data are presented as the mean (SD)*.

* *he data of polymyxin B data were only available in the Sorin circuit; therefore, they had no standard deviation*.

† *og P and protein-binding rate for the individual drugs were obtained from DrugBank^®^, a web-accessible public database*.

### The Difference in Drug Loss Between the Maquet Circuit and the Sorin Circuit

The drug recovery rates of tigecycline, caspofungin, meropenem, and cefoperazone in these two circuits were similar. In the Sorin circuit, significant drug loss occurred in teicoplanin, and a small amount of drug loss occurred in sulbactam, while in the Maquet circuit, the drug recovery rates of teicoplanin (*P* = 0.094) and sulbactam (*P* = 0.105) were not significantly different from those in the control group. Voriconazole showed significant drug loss after 3 h of operation in the Maquet circuit, while it remained unchanged in the first 3 h of operation in the Sorin circuit. The recovery rate of voriconazole at 24 h was 53% in the Maquet and 78% in the Sorin circuit.

### Correlation Between Drug Recovery and Log P or Protein Binding Rate

The relationship between drug recovery and lipophilicity (represented as log P) or protein binding rate was analyzed using linear regression. The log P, protein binding rate, and average drug recovery rates of all drugs were summarized in Table 1. The correlation between log P of drugs and the extent of their loss in the blood-primed circuit at 24 h was not significant (*r*^2^ = 0.008, *P* = 0.225), nor was the protein binding rate of drugs (*r*^2^ = 0.168, *P* = 0.479).

## Discussion

To the best of our knowledge, this is the first *ex vivo* experiment to evaluate the sequestration of teicoplanin, tigecycline, polymyxin B, cefoperazone, and sulbactam in ECMO circuits.

Teicoplanin showed a large loss in the Sorin circuit (59%), which may due to its high protein binding rate (90–95%). Similar to our findings, Chen et al. (15, 16) recommended four doses of teicoplanin administered within the initial 72 h at a dose of 12 mg/kg/dose, a higher than the normal dose, which could successfully achieve a therapeutic serum trough concentration of teicoplanin (>10–15 mg/L). Previous studies have also pointed out that critically ill patients who did not receive ECMO support also need to increase the dosage of teicoplanin: compared with patients receiving lower loading dose (6 mg/kg/ dose, 4 doses), critically ill patients receiving high loading dose of teicoplanin (12 mg/kg/ dose, 4 doses) are more likely to reach sufficient blood concentration (17). Combined with the above studies and the results of this experiment, the drug loss of teicoplanin during ECMO support may be the result of the drug adsorption by the ECMO circuit and the pathophysiological changes caused by critical diseases. Therefore, we suggest that patients receiving Sorin ECMO support should increase the dosage of teicoplanin to ensure the therapeutic effect. It is worth noting that there is almost no drug loss of teicoplanin in the Maquet circuit, which may be related to the differences of membrane oxygenator and PVC pipeline coating between the two types of ECMO. However, there are no other studies to compare the difference of teicoplanin drug loss in these two types of ECMO. More *ex vivo* and *in vivo* experiments are needed to guide the administration of teicoplanin during ECMO support.

At present, only one study has reported that ECMO has no effect on tigecycline pharmacokinetics (18). Similar to this case report, no drug loss of tigecycline was observed in the Maquet/Sorin circuit in our study, which may be due to its weak lipophilicity (log P 0.8). The average Vd of tigecycline in critically ill patients is 398 L, which is therefore unlikely to be noticeably increased simply by dilution into the system. Present studies have shown that ECMO does not affect the pharmacokinetic parameters of tigecycline. However, researches conducted in critically ill patients have recommended a high-dose tigecycline regimen (LD 200 mg, MD 100 mg, BID) (19, 20). Therefore, it is suggested that the plasma concentration of tigecycline be monitored regularly during ECMO support to prevent the failure of anti-infection treatment.

We detected a significant drug loss of polymyxin B in the Sorin circuit at 6 h. The drug recovery was 74% in the Sorin circuit group and 93% in the control group. Unexpectedly, the drug recovery in the Sorin circuit and the control group was 62 and 60% at 24 h, respectively. Polymyxins are highly surface-active; therefore, their drug loss from aqueous solutions onto the surfaces of the apparatus used during the collection and processing of samples may have an impact on recovery (21). Since we experimented with the Sorin circuit only once, we hypothesized that the recovery rate of polymyxin B at 24 h in the control group (60%) might be reduced due to its adherence to the collection device during processing. The results of our study need to be confirmed by more experiments with a large sample size.

Cefoperazone-sulbactam is a hydrophilic drug, which makes the sequestration of Cefoperazone-sulbactam in the ECMO circuit less likely than that of lipophilic drugs. In our study, Cefoperazone-sulbactam showed slight drug loss after 24 h of ECMO operation, further *in vivo* experiments are needed to figure out whether clinical dosage needs to be adjusted during ECMO operation.

No significant drug sequestration of caspofungin was observed in this study, the average drug recovery at 24 h was 83 and 85% in the ECMO circuit and control group, respectively. This is contrary to other *ex vivo* and *in vivo* experiments. An *ex vivo* experiment conducted by Shekar et al. found that the average drug recovery of caspofungin at 24 h in the ECMO circuit was 56% (6). A case report (22) observed that the standard dose of caspofungin failed to reach the target plasma concentration level during ECMO support. However, other *in vivo* studies (23–25) have suggested that ECMO does not affect the pharmacokinetic characteristics of caspofungin. Caspofungin is hydrophilic (log P −2.798) but has a high protein binding rate (97%), which may lead to significant differences in its recovery in different types of ECMO circuits. Given the large variation among patients and the extremely limited sample size of the above studies, it is difficult to draw a unified conclusion. Therefore, the dose of caspofungin during ECMO support still needs to be adjusted according to the monitoring results of plasma concentration.

Similar to caspofungin, micafungin is hydrophilic (log P −1.5) and has a high protein binding rate (>99%). An *ex vivo* study conducted by Watt et al. (26) showed that the average drug recovery of micafungin in the ECMO loop was 26–43% at 24 h, compared to 57% in the control group. Watt explained that drug degradation is the most likely mechanism of loss in the controls. Micafungin is known to degrade in light, neither the ECMO circuit nor the control group was light-avoiding, which might lead to a large amount of degradation of micafungin. However, in our study, the drug recovery of micafungin was 67% in the Sorin loop and 99% in the control group at 24 h, which was much higher than the results of Watt's research. Therefore, the drug degradation of micafungin may not explain its significant drug loss in the ECMO circuit. *In vivo* studies found that in infants on ECMO, the Vd of micafungin was 20–90% higher than that reported in infants not on ECMO (27). However, a prospective observational study carried out in 12 adult patients on ECMO found no significant changes in the pharmacokinetic parameters of micafungin (28). Infants have less blood volume than adults, so ECMO circuits might have a greater effect on the Vd of micafungin in infants. Both *ex vivo* and *in vivo* studies in infants have shown remarkable drug loss of micafungin during ECMO support, therefore, we recommend increasing the dose of micafungin in infants on ECMO. As for adult patients on ECMO, we could maintain the conventional dose and adjust the dose regimen of micafungin according to the plasma concentration.

Previous *ex vivo* experiments have shown that the drug recovery of voriconazole at 24 h in the ECMO circuit was only 29% (5). This study detected an average 24 h recovery of 60% for voriconazole in the ECMO circuit, which also showed significant drug loss. Consistent with the results of *ex vivo* experiments, *in vivo* experiments also showed insufficient plasma concentrations of voriconazole in patients under ECMO. Plasma concentration monitoring of voriconazole in two adult patients under ECMO showed that more than 50% of the measured plasma concentration levels were below the detection lower limit (5). Existing researches have shown that due to the high lipophilicity of voriconazole (log P 2.561), substantial drug loss of voriconazole occurs during the ECMO process, requiring a routine increase in the dose of voriconazole. It is worth noting that indiscriminately increasing the dose of voriconazole may cause its plasma concentration to exceed the treatment window and lead to adverse events (23). Therefore, in the treatment of voriconazole during ECMO support, the peak concentration and trough concentration should be closely monitored at the same time.

The sequestration of meropenem in ECMO circuits in our research was comparable to previous reports. Consistent with previous reports [80% loss at 24 h (9); 17% loss at 3 h (9)], the average meropenem loss at 24 h in the circuits was 55% in our study. The drug loss of meropenem can be attributed to its instability at physiological temperature. Patrick suggested that optimization of meropenem treatment during ECMO requires either more frequent dosing, a dose increase, or prolonged infusion due to its degradation and significant sequestration in the ECMO circuit after 4–6 h of treatment (29). However, in a case-control study conducted by Donadello et al., ECMO therapy did not significantly influence meropenem pharmacokinetics compared with well-matched non-ECMO controls (30). Another 2 studies (31, 32) also pointed out that in patients receiving meropenem on ECMO, standard dosing (1 g 8 h) should achieve routinely targeted plasma concentrations. However, incremental dosing or continuous infusion may be needed when targeting higher plasma concentrations and/or in patients with elevated creatinine clearance.

Previous studies have shown that different types of pumps and circuits affect drug sequestration during ECMO therapy. Wildschut et al. (10) found that the recovery of midazolam and fentanyl in centrifugal pump circuits with hollow-fiber membrane oxygenators was significantly higher than that in neonatal roller pump circuits with silicone membranes. According to Park's research (33), the tubing material could be the source of the cause of drug loss rather than the coating material used for the ECMO circuit. The difference between Maquet and Sorin ECMO circuits is the surface coating material. Maquet is coated with Bioline (an albumin-heparin coating in which heparin is covalently bonded to albumin immobilized on the surface), and Sorin is coated with choline phosphate. Teicoplanin, which has low lipophilicity, lost far more Sorin than Maquet circuits. Therefore, when the tubing material is the same, the coating material will become the primary cause of drug loss in ECMO circuits, which is associated with drug lipophilicity.

Simple linear regression did not find any significant correlation between log P (*r*^2^ = 0.008, *P* = 0.225) or protein binding rate (*r*^2^ = 0.168, *P* = 0.479) of drugs and the extent of their loss in the blood-primed circuit at 24 h. We failed to find their correlation using non-linear regression analysis. Shekar et al. declared that drugs with significantly reduced concentrations at 24 h were either highly protein-bound (>80%), highly lipophilic (log P > 2.3), or both. However, in our research, the concentration of highly protein-bound drugs, such as cefoperazone, remained relatively stable after 24 h of circulation; drugs with a low protein-binding rate and low lipophilicity, such as sulbactam and meropenem, showed important losses in ECMO circuits. More research is needed on these drugs to understand their adsorption in ECMO circulation.

Our *ex vivo* study has some limitations. First of all, due to the high cost of the ECMO equipment, we only conducted one experiment for each type of ECMO circuit, the solidity of the results might suffer from too few replicates of the experiment. More replicates on these drugs are needed in the future to clarify the influence of the ECMO circuit on them. Secondly, the concurrent presence of 9 physically compatible drugs in the circuit and control groups may have had an impact on competitive binding to plasma proteins or the circuit components, thereby influencing the results. And lastly, a reservoir bag was necessary to construct a bypass to maintain pressure on the circuit, which may have influenced the circuit drug loss because of its own drug absorption. Similarly, the drug lost in the control groups due to the binding of drugs to the polypropylene tubes was immeasurable.

In conclusion, in the two ECMO circuits, meropenem and voriconazole were significantly lost, cefoperazone was slightly lost, while tigecycline and caspofungin were not lost. Drugs with high lipophilicity were lost more in the Maquet circuit than in the Sorin circuit. This study needs more *in vivo* studies with larger samples for further confirmation, and it suggests that therapeutic drug concentration monitoring should be strongly considered during ECMO.

## Data Availability Statement

The original contributions presented in the study are included in the article/Supplementary Material, further inquiries can be directed to the corresponding author/s.

## Ethics Statement

The studies involving human participants were reviewed and approved by the Medical Ethics Committee of NanFang Hospital of Southern Medical University (NFEC-2020-021). The patients/participants provided their written informed consent to participate in this study.

## Author Contributions

ZC designed and coordinated the study. YZ and HH collected and analyzed data and developed the manuscript for publication. QO and TS assisted with the operation of ECMO circuits. QZ guided antimicrobial dosage. HZ was in charge of the blood collection from volunteers. JW and ZZ assisted with statistical analysis. JL was responsible for the measurement of drug concentration. All authors provided final approval of the version submitted for publication.

## Funding

This work was supported by the Natural Science Foundation of China (Grant Number 81871604); the Clinical Drug Research Foundation of Guangdong Province, China (Grant Number 2020ZJ04).

## Conflict of Interest

The authors declare that the research was conducted in the absence of any commercial or financial relationships that could be construed as a potential conflict of interest.

## Publisher's Note

All claims expressed in this article are solely those of the authors and do not necessarily represent those of their affiliated organizations, or those of the publisher, the editors and the reviewers. Any product that may be evaluated in this article, or claim that may be made by its manufacturer, is not guaranteed or endorsed by the publisher.
