# Supplementary material for: Effects of ex vivo Extracorporeal Membrane Oxygenation Circuits on Sequestration of Antimicrobial Agents
Source: Front Med (Lausanne). 2021 Dec 1;8:748769. doi: 10.3389/fmed.2021.748769 (PMC8671752; doi:10.3389/fmed.2021.748769)
Supplement: Supplementary file 1 [file Data_Sheet_1.DOCX]

**Additional file 1. Materials for the Maquet and Sorin ECMO circuits.**

| **Component** | **Manufacturer** | **Model** | **Material** |
| --- | --- | --- | --- |
| Oxygenator | Maquet | Adult Quadrox PLS^*^ | Polymethyl pentane hollow fibers with Bioline^†^ coating |
| Pump | Maquet | Centrifugal pump | Polycarbonate |
| Tubing | Maquet | PLS^*^ tubing | Bioline^†^-coated polyvinylchloride |
| Cannula | Maquet | Arterial cannula | Bioline^†^-coated polyvinylchloride |
| Oxygenator | Sorin | D905 EOS ECMO | Polymethyl pentane hollow fibers with phosphorylcholine coating |
| Pump | Sorin | Revolution 5 centrifugal pump | Polycarbonate |
| Tubing | Sorin | Smart tubing | Phosphorylcholine-coated polyvinylchloride |
| Cannula | Sorin | Arterial cannula | Phosphorylcholine-coated polyvinylchloride |

^*^PLS: Permanent life support.

^†^Bioline coating: heparin + recombinant human albumin.
