# Supplementary material for: Effects of ex vivo Extracorporeal Membrane Oxygenation Circuits on Sequestration of Antimicrobial Agents
Source: Front Med (Lausanne). 2021 Dec 1;8:748769. doi: 10.3389/fmed.2021.748769 (PMC8671752; doi:10.3389/fmed.2021.748769)
Supplement: Supplementary file 2 [file Data_Sheet_2.DOCX]

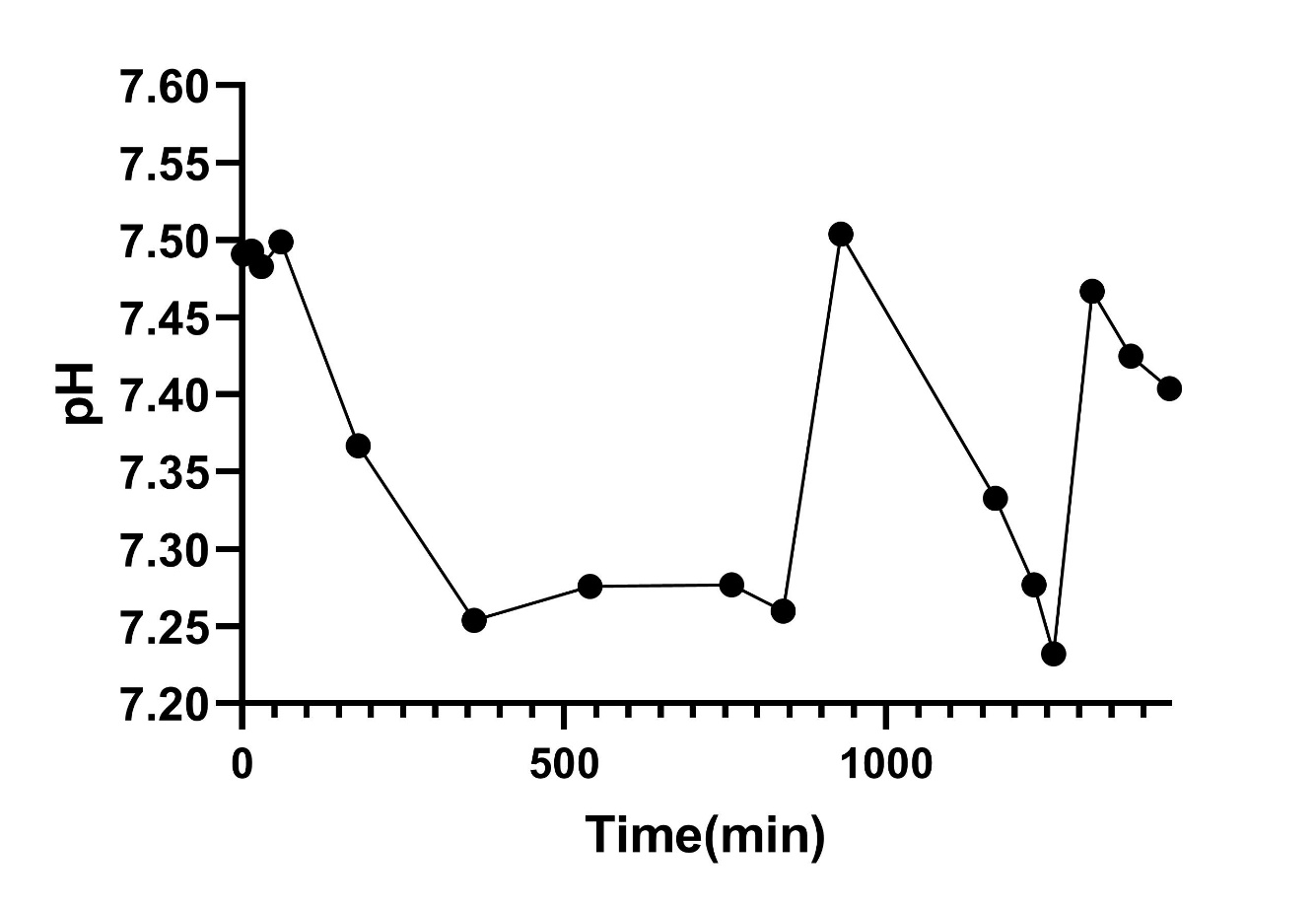
**Additional file 5. The pH value in ECMO circuits over the 24 h.**

The pH value in the individual circuits over the 24 h was between 7.226 and 7.504.
