# Supplementary material for: Effects of ex vivo Extracorporeal Membrane Oxygenation Circuits on Sequestration of Antimicrobial Agents
Source: Front Med (Lausanne). 2021 Dec 1;8:748769. doi: 10.3389/fmed.2021.748769 (PMC8671752; doi:10.3389/fmed.2021.748769)
Supplement: Supplementary file 3 [file Data_Sheet_3.DOCX]

**Table 3. Mean drug recovery for each drug at different time points in Maquet circuits (n=2).**

| **Parameter** | **Teicoplanin** | **Meropenem** | **Cefoperazone** | **Sulbactam** |
| --- | --- | --- | --- | --- |
| 2 min | 100% (0%) | 100% (0%) | 100% (0%) | 100% (0%) |
| 5 min | 97% (2%) | 100% (0%) | 102% (3%) | 100% (0%) |
| 15 min | 98% (2%) | 100% (1%) | 100% (4%) | 95% (5%) |
| 30 min | 100% (5%) | 101% (4%) | 100% (2%) | 92% (11%) |
| 1 h | 102% (3%) | 98% (0%) | 102% (1%) | 98% (2%) |
| 3 h | 93% (10%) | 93% (2%) | 98% (2%) | 94% (1%) |
| Control-3 h^*^ | 101% (5%) | 97% (4%) | 100% (0%) | 92% (4%) |
| 6 h | 91% (8%) | 83% (7%) | 99% (1%) | 63% (40%) |
| Control-6 h^*^ | 96% (0%) | 91% (9%) | 99% (0%) | 65% (38%) |
| 12 h | 86% (5%) | 64% (2%) | 91% (5%) | 63% (28%) |
| Control-12 h^*^ | 88% (5%) | 77% (8%) | 100% (2%) | 85% (10%) |
| 24 h | 80% (13%) | 45% (5%) | 81% (1%) | 74% (9%) |
| Control-24 h^*^ | 83% (14%) | 65% (10%) | 98% (2%) | 81% (9%) |

^*^Control-3 h represents the results at 3 h in the control groups.

Data are presented as the mean (SD).
