# Supplementary material for: Effects of ex vivo Extracorporeal Membrane Oxygenation Circuits on Sequestration of Antimicrobial Agents
Source: Front Med (Lausanne). 2021 Dec 1;8:748769. doi: 10.3389/fmed.2021.748769 (PMC8671752; doi:10.3389/fmed.2021.748769)
Supplement: Supplementary file 4 [file Data_Sheet_4.DOCX]

**Table 4. Drug recovery for each drug at different time points in the Sorin circuit.**

| **Parameter** | **Teicoplanin** | **Meropenem** | **Cefoperazone** | **Sulbactam** | **Polymyxin B** |
| --- | --- | --- | --- | --- | --- |
| 2 min | 100% | 100% | 100% | 100% | 100% |
| 5 min | 103% | 104% | 100% | 98% | 118% |
| 15 min | 106% | 104% | 100% | 99% | 86% |
| 30 min | 104% | 104% | 99% | 99% | 84% |
| 1 h | 105% | 103% | 102% | 100% | 104% |
| 3 h | 108% | 100% | 103% | 101% | 117% |
| Control-3 h^*^ | 88% | 97% | 103% | 102% | 91% |
| 6 h | 97% | 92% | 98% | 96% | 74% |
| Control-6 h^*^ | 95% | 102% | 98% | 97% | 93% |
| 12 h | 72% | 74% | 92% | 92% | 74% |
| Control-12 h^*^ | 120% | 106% | 101% | 99% | 105% |
| 24 h | 41% | 47% | 75% | 77% | 62% |
| Control-24 h^*^ | 101% | 95% | 98% | 97% | 60% |

^*^Control-3 h represents the results at 3 h after ECMO administration in the control groups.
